# Application study of surgical instruments information management system in sports medicine specialty

**DOI:** 10.1038/s41598-024-56809-5

**Published:** 2024-03-14

**Authors:** Jin Tang, Min Zhuang, Ping Lin, Zichen Wang, Jinzhong Zhao

**Affiliations:** 1 The Operating Theater, Shanghai Sixth People’s Hospital, Shanghai Jiao Tong University School of Medicine, 600 Yishan Road, Shanghai, China; 2 The Department of Sports Medicine, Shanghai Sixth People’s Hospital, Shanghai Jiao Tong University School of Medicine, Shanghai, China

**Keywords:** Surgical instruments management, Operating room, Information system, Sports medicine, Health care, Health occupations

## Abstract

The management of surgical instruments is related to the safety and efficiency of surgical operations, and a surgical instruments information management system (SIIMS) has been developed. The aim of the current study is to explore the application value of the SIIMS in sports medicine specialty. A set of self-developed SIIMS for sports medicine surgeries was applied to the study. The application value of the SIIMS was verified by comparing the safety and efficiency of instrument manipulation before and after its application, with instrument accidents, instrument repair rate, instrument scrap rate and instrument use efficiency as indicators. Through the application of the SIIMS, the incidence of surgical instrument accidents decreased from 3.7 times to 1.8 times (P = 0.02), the number of instrument repair decreased from 7.7 times to 2.9 times (P = 0.00), and the number of scrapped instruments decreased from 5.1 to 2.3 (P = 0.03), when referred to per thousand operations. Before and after the application of the SIIMS, the average instrument use efficiency was 74.0% ± 3.3% and 88.2% ± 4.4%, respectively, with statistically significant difference (P = 0.00). The application of the SIIMS in sports medicine specialty is helpful to the fine management of surgical instruments, improve surgical safety and instrument use efficiency.

Surgery, especially orthopedic surgery is highly dependent on medical equipment. The management of surgical instruments and equipment is related to the safety and efficiency of surgical operations. The smooth operation depends on the timely, accurate provision and safe and effective use of surgical instruments. Delayed, inadequate, or incorrect provision of medical equipment, as well as instrument failure during surgery can cause surgery to be delayed or impossible^1–4^. From another point, improper use and handling of medical devices can cause damage to the equipment, which endangers surgical operations in turn, and results in loss of medical assets.

Sports medicine is a rapidly developing orthopedic specialty, in which various minimally invasive repair and reconstruction techniques rely on a large number of advanced fine surgical instruments. The reasonable configuration, operation and safe and effective use of these instruments are of great importance both to clinical practice and to extend their service lives^5,6^. In recent years, the refinement and information management of hospital medical instrument, especially surgical instrument, is an important development trend^7–10^. However, to our knowledge, there is no international study on the application of surgical instruments information management system (SIIMS) in sports medicine specialty.

Therefore, the purpose of this study is generally to explore the application value of the SIIMS in sports medicine specialty. Because the application value of the SIIMS manifests in many aspects of the service life of the surgical instruments, in the current study we focused mainly on its value on the extension of service life of surgical instrument, from the aspects of instrument accidents, instrument repair rate, instrument scrap rate and instrument use efficiency.

The current study is novel in that it was the first study to evaluated the application value of SIIMS in sports medicine specialty, specially form the aspects of the value on the extension of the service lives of the instruments. It was also the first study that revealed that the application of a surgical instrument tracking system may influence the instrument user’s behavior.

## Methods

This was a retrospective analysis of prospective collected research data. From 2015 through 2021, we carried out a study on the application value of informatization in the precision management of surgical instruments in sports medicine specialty. The research object of the current study were the surgical instruments in the sports medicine department of our institution, from the aspects of their service life. The intervening measure was the application of the SIIMS. This study was not related to experiments on humans and/or the use of human tissue samples, human information and data. According to the Approaches to Ethical Review of Life Sciences and Medical Research Involving Humans jointly issued by the Chinese National Health Commission, the Ministry of Education, the Ministry of Science and Technology and the National Administration of Traditional Chinese Medicine, there was neither need for ethics approval from the institutional review board of Shanghai Sixth People’s Hospital nor informed consent from the patients. All methods were carried out in accordance with relevant guidelines and regulations of the institution regarding the configuration, storage, transportation, use, maintenance, repair and scrap of medical equipment.

From 2015 through 2017, we collected the basic data of instrument management in the operating room of sports medicine specialty in Shanghai Sixth People's Hospital, discussed the crux of instrument management, and determined the technical indicators and basic design scheme of the SIIMS. In 2018, we developed, preliminarily verified and improved a software system (with a software copyright registration number 2019SR1310456) suitable for the information management of surgical instruments in sports medicine specialty. From 2019 to 2021, this SIIMS was applied, and further verified according to the technical indicators set before the study began. Our hypothesis is that the application of the SIIMS in sports medicine specialty will increase surgical safety and improve the efficiency of the use of instruments.

The SIIMS is based on the client/server (C/S) architecture design, mainly composed of the server, client and interface management. The client mainly includes functional modules for the management of instrument configuration, use, maintenance, and scrap, user management and statistical analysis. The interface management defines the interface with the handheld scanning instrument. The establishment of the platform database is a collection of electronic files of all instruments during their useful life cycle. The system includes functions such as login authentication, query, statistical report, system extension, exogenous instrument management and instrument disinfection management. The electronic file of a single instrument in its life cycle is set up and updated in real time in four modules, namely, the in-configuration state, the in-use state, the in-maintenance state and the scrapped state. The administrator is responsible for the information input of the corresponding modules according to the authority given.

In our institution, the disinfection of surgical instruments in sports medicine specialty is mainly completed on the spot in the operating room, and the disinfection status can be known immediately. In some cases, when the on-site disinfection is full, some instruments are sent to the central supply room of the hospital for disinfection. At this time, the disinfection status of these instruments is tracked. In the expanded function of this system, there is an instrument disinfection management plate, which gives the responsible personnel of the central supply room the corresponding administrator rights, through the interface management and the scanning gun of the central supply room, to enter the information of the disinfection and transfer status of this part of the instrument (acceptance, in disinfection, disinfection completed, transport)^11^.

The technical means of this study are mainly to compare and analyze the safety and effectiveness of the instrument use before (2015–2017) and after (2019–2021) the application of the SIIMS. The specific indicators include the accident rate of instrument manipulation, the repair rate, the scrap rate, and the use efficiency of the instruments.

Instrument manipulation accident refers to the intraoperative accidental damage during the operation of related instruments and the need for temporary instruments replacement, such as serious damage to the arthroscope, fracture of the nucleus pulposus clamp, broken teeth of the free body clamp, broken hook of the suture hook etc. Instrument repair refers to the repair of faulty instruments to retore their function, such as poor contact of the camera cable plug, poor contact of the power tool battery case, etc., and instrument failure caused by improper operation such as mild damage to the arthroscope. Instrument scrap refers to the failure of the instrument that cannot be resolved by maintenance and its use must be abandoned. Instrument accident rate, instrument repair rate and instrument scrap rate are based on the statistics of the whole sports medicine department of our institution and are calculated with the current instruments in use and every 1000 operations as references, respectively.

Instrument use efficiency analysis was conducted by taking the use of arthroscopes as a representative. The number of operations completed with each arthroscope in the life cycle (use—maintenance—use, until scrapped) was counted, and the use efficiency of up to 1000 operations was set as 100%. Instrument use efficiency analysis was carried out for surgeons who worked full time in 2015 through 2017 and 2019 through 2021. In the sports medicine department of our institution, each principal surgeon could only use the arthroscope assigned to him, and the utilization rate was calculated based on the number of operations that the arthroscope had been used during the time period when each surgeon scrapped the arthroscope. If the surgeon had multiple arthroscopes scrapped during that time period, the average was calculated based on the use efficiency of each scrapped arthroscope as a representative. If the surgeon had no scrapped arthroscopes during that time period, he was not included in the statistics.

Statistical analysis was performed on relevant data with suitable conditions (SPSS-22), and P < 0.05 was used as the standard for statistical significance. For the enumeration data, ANOVA was carried out, the measurement data was tested for normality first, the data conforming to normal distribution was analyzed by Student t test, and the data not conforming to normal distribution was analyzed by Chi-square test.

## Results

In the two target time periods, the number of surgeons in the sports medicine department of our institution was 11 and 15, respectively, while the average number of surgical nurses in the sports medicine specialty (including scrub nurses and circulating nurses, who were involved in various manipulations, such as rinsing, drying, packing and disinfection of the instruments) was 7 and 14, respectively. In both time periods, the number of operations gradually increased year by year, from 3267 in 2015 to 6682 in 2021. The number of operating instruments increased from 1333 in 2015 to 1643 in 2021 (Table 1).

**Table 1:** Composition of medical care, amount of operation and number of operating instruments before and after the application of the SIIMS.

	Pre-application			Post-application		
	2015	2016	2017	2019	2020	2021
Surgeons	11	11	11	15	15	15
Specialist nurses	6	7	8	13	14	15
Amount of surgery	3267	3583	4154	5639	5872	6682
No. of running instruments	1333	1364	1396	1475	1530	1643

Before and after the application of the SIIMS, the number of instrument accidents decreased, with an average of 13.7 and 11 cases per year, respectively (P = 0.0.32). The proportion of instrument accidents in the total operating instruments decreased to 10.1‰ and 6.7‰ respectively (P = 0.09). The incidence of instrument accidents per 1000 operations was significantly reduced, from an average of 3.7 to an average of 1.8 (P = 0.02) (Table 2).

**Table 2:** The incidence of instrument accidents before and after the application of the SIIMS.

	Pre-application			Mean	Post-application			Mean	P-value
	2015	2016	2017		2019	2020	2021		
Absolute value	11	17	13	13.7	10	9	14	11	0.32
‰	8.2	12.5	9.5	10.1	5.7	5.9	8.5	6.7	0.09
Referred to per thousand surgeries	3.4	4.7	3.1	3.7	1.8	1.5	2.1	1.8	0.02

Before and after the application of the SIIMS, the number of instrument repair decreased significantly, from an average of 28.3 to an average of 17.7 times per year, respectively (P = 0.01). The proportion of repaired instrument to the total operating instrument decreased, from an average of 17.4‰ to an average of 11.9‰ (P = 0.05). The number of instrument repair referred to 1000 operations decreased significantly, from an average of 7.7 to an average of 2.9 times (P = 0.00) (Table 3).

**Table 3:** Incidence of instrument repair before and after the application of the SIIMS.

	Pre-application			Mean	Post-application			Mean	P-value
	2015	2016	2017		2019	2020	2021		
Absolute value	25	27	33	28.3	16	18	19	17.7	0.01
‰	18.8	19.8	13.6	17.4	10.8	11.8	13.0	11.9	0.05
Referred to per thousand surgeries	7.7	7.5	7.9	7.7	2.8	3.1	2.8	2.9	0.00

Before and after the application of the SIIMS, the annual number of scrapped instruments decreased from an average of 19 to an average of 14.3 pieces per year (P = 0.22). The proportion of scrapped instruments in total operating instruments decreased from 13.9‰ to 9.8‰ (P = 0.17). The number of scrapped instruments per 1000 operations decreased significantly, from an average of 5.1 to an average of 2.3 (P = 0.03) (Table 4).

**Table 4:** Scrap rate of instruments before and after the application of the SIIMS.

	Pre-application			Mean	Post-application			Mean	P-value
	2015	2016	2017		2019	2020	2021		
Absolute value	14	24	19	19	12	14	17	14.3	0.22
‰	10.5	17.6	13.6	13.9	8.1	9.2	11.6	9.8	0.17
Per thousand procedures	3.9	6.7	4.6	5.1	2.1	2.4	2.5	2.3	0.03

Before the application of the SIIMS, the average instrument use efficiency was 74.0% ± 3.3% (n = 7). After the application of the SIIMS, the average instrument use efficiency was 88.2% ± 4.4% (n = 9). The increase of instrument use efficiency was statistically significant (P = 0.00).

## Discussion

This study shows that the application of SIIMS in sports medicine specialty can significantly reduce the incidence of surgical instrument manipulation accidents, reduce the rate of surgical instrument repair and scrap per thousand operations, and increase the efficiency of surgical instruments.

The sports medicine specialty surgery is characterized by minimally invasive arthroscopic surgery, and the surgical instruments are mainly arthroscopes and endoscopic operating instruments. As an observation instrument, the arthroscope is easily damaged by mechanical impact, friction or scalding. The instruments operated under arthroscope are mainly thin instruments and open instruments, which are more likely to be damaged theoretically. Such damage may be caused by improper operation during the operation, or by accidents during cleaning, packing and disinfection. In general, the first reason for the loss of sports medicine instrument is natural aging, which leads to poor contact of camera cable plug, optical cable transmittance loss, poor contact of power tool battery box, blunt mouth of basket forceps, and closure barrier of suture retriever and etc. Another part of the reason is mainly related to improper operation, which leads to arthroscope damage, suture passing hook breakage, nucleus pulposus clamp breakage, free body clamp breakage and so on. Sports medicine surgeons and operating room nurses are the main operators of the instruments.

The purpose of the development and application of the SIIMS is the fine management of medical instruments, in order to use the SIIMS to increase the efficiency of medical instrument configuration, promote the standardization of the use of medical instruments, strengthen the responsibility of protection and maintenance, improve the efficiency and quality of maintenance. Therefore, we have developed evaluation indicators for the different associated objects in the stage of instrument configuration, use and maintenance. In the instrument use stage, it is mainly to clarify the management objectives of instrument use and maintenance personnel and implement the responsibility. All relevant personnel will be reminded that the instrument configuration, use and maintenance status will be counted and associated with individuals. This is a reminder of responsibility, and no disciplinary action is required. In this study, the safety and efficiency of instruments that require repeated use were mainly evaluated.

However, this study found that, on the whole, the use of sports medicine instrument was relatively safe, and its use efficiency was quite high. In this study, before and after the application of the SIIMS, the incidence of instrument accidents in every 1000 operations was only 3.7 and 1.8 times respectively, the average number of surgical instrument maintenance was only 7.7 and 2.9 times, and the average number of instrument scrap was only 5.1 and 2.3. Each arthroscopic arthroscope could be used for an average of 740 and 882 operations before and after the application of the SIIMS. However, the pursuit of surgical safety and instrument efficiency should never end.

The results of this study are very interesting. Although the SIIMS is not linked to rewards and punishments, it does serve as a reminder for instrument manipulation personnel to pay attention to the safety and to increase the efficiency of instrument use. The SIIMS seems to make the relevant personnel aware of the responsibility and obligation to protect the instrument when manipulating them. In fact, the SIIMS also brings benefits in terms of instrument configuration management and maintenance, just outside the focus of this study.

The information management of medical instruments is an important part^12–16^ of modern hospital management. The information management of surgical instruments in sports medicine specialty is conducive to each functional department's understanding and control of the application status of specialized instruments and equipment, is beneficial to the reasonable allocation and efficient and safe use of instrument and equipment and can bring benefits to the overall precision and modern management of sports medicine operating room. Whether this system can be popularized and applied in the management of surgical instruments in other specialties remains to be further discussed^17,18^.

There are some limitations in this study. First of all, the instruments used in the second phase were not brand new, and most of them were used in the first phase. The safety indicators, the repair and scrap rates, and use efficiency of the instruments used in the second phase were actually related to the first phase, and the indicators of the first phase were related to the use status of the instruments before the study began. Theoretically, in terms of the evaluation of the use of the instrument, it is possible to avoid these interferences by evaluating only the newly configured instruments. However, because the number of newly added instruments is not large in the target periods of the study, it would have been difficult to carry out relevant research, so all the instruments in use were used as the study object. In addition, the improvement of instrument safety and efficiency may be related to the improvement of instrument use proficiency. In the period after the application of the SIIMS, surgeons might have more experience in using the instrument than before the application of the SIIMS. However, there was no significant change in the efficiency and safety of instrument use during each time period, suggesting that improvement in proficiency was not the most important associated factor.

### Conclusion

The application of the surgical instrument information management system in sports medicine specialty is helpful to the fine management of surgical instruments, improve surgical safety and instrument use efficiency.

## Data Availability

The datasets used the current study are available from the corresponding author (J.T.) on reasonable request.
